# Cloning, expression and characterization of gE protein of Duck plague virus

**DOI:** 10.1186/1743-422X-7-120

**Published:** 2010-06-08

**Authors:** Hua Chang, Anchun Cheng, Mingshu Wang, Dekang Zhu, Renyong Jia, Fei Liu, Zhengli Chen, Qihui Luo, Xiaoyue Chen, Yi Zhou

**Affiliations:** 1 Avian Diseases Research Center, College of Veterinary Medicine of Sichuan Agricultural University, Yaan, Sichuan, 625014, China; 2 Key Laboratory of Animal Diseases and Human Health of Sichuan Province, Yaan, Sichuan, 625014, China; 3 Epizootic Diseases Institute of Sichuan Agricultural University, Yaan, Sichuan, 625014, China

## Abstract

**Background:**

The gE protein of duck plague virus is the important membrane glycoprotein, its protein characterization has not been reported. In this study, we expressed and presented the characterization of the DPV gE product.

**Results:**

According to the sequence of the gE gene, a pair of primers were designed, and the DNA product with 1490bp in size was amplified by using the polymerase chain reaction (PCR). The PCR product was cloned into pMD18-T vector, and subcloned into pET32a(+), generating the recombinant plasmid pET32a/DPV-gE. SDS-PAGE analysis showed that the fusion pET32a/DPV-gE protein was highly expressed after induction by 0.2 mM IPTG at 30°C for 4.5 h in Rosseta host cells. Over expressed 6×His-gE fusion protein was purified by nickel affinity chromatography, and used to immunize the rabbits for the preparation of polyclonal antibody. The result of the intracellular localization revealed that the gE protein was appeared to be in the cytoplasm region. The real time PCR, RT-PCR analysis and Western blotting revealed that the gE gene was produced most abundantly during the late phase of replication in DPV-infected cells.

**Conclusions:**

In this work, the DPV gE protein was successfully expressed in a prokaryotic expression system, and we presented the basic properties of the DPV gE product for the first time. These properties of the gE protein provided a prerequisite for further functional analysis of this gene.

## Background

Duck plague (DP), which is caused by DPV, is an acute, febrile, contagious, and septic disease of waterfowl (ducks, geese, and swans) [1]. DPV has been classified as belonging to the Alphaherpesvirinae subfamily of the family Herpesviridae on the basis of the report of the Eighth International Committee on Taxonomy of Viruses (ICTV), but it has not been grouped into any genus [2]. The genome of DPV, a linear and double stranded DNA, is about 150 kb. Recently, an increasing number of DPV genes, such as UL5 [3], UL6 [4], UL22, UL23(TK) [5], UL24 [5,6], UL25, UL26, UL26.5, UL27, UL28, UL29, UL30 [7], UL31 [8,9], UL32, UL33, UL34 [10], UL35 [8,11], UL44 (gC) [12], UL50 [13], UL51 [14], US8 [10], US2 and US10 [15] have been identified. Some genes were not essential for replication of the virus in cell culture in Herpesviridae, these dispensable gene products were, however, thought to be important for virus growth and spread in the natural host [16]. The envelope glycoprotein E (gE) in Herpesviridae was important for the expression of virulence of the virus. It was necessary that the virus transfered in olfactory, trigeminal, sympathetic, and parasympathetic pathways [17,18], and played an important role in cell-to-cell spread, though it was not a essential protein for in vitro replication [19-21]. In addition, the gE protein, an important envelope glycoprotein, was present in almost all examined the field isolates, and the gE antigen was used in the serological diagnosis, which was detected the antibodies against gE in the natural infection [22].

In 2006, a DPV genomic library was successfully constructed in our laboratory [23]. Sequence analysis showed that the gE gene of DPV was predicted to encode a 490 amino acid protein with a molecular mass of 54 kDa [10]. The report focused on the product of the DPV gE gene. We constructed the recombinant expression vector pET32a/DPV-gE, the fusion pET32a/DPV-gE protein (approximately 74 kDa) was expressed by the addition of isopropyl-β-D-thiogalactopyranoside (IPTG). The recombinant gE protein was purified and used to immunize the rabbits for the preparation of polyclonal antibody. We examined further the intracellular localization of the gE protein using the rabbit polyclonal antiserum specific to it in DPV-infected cells. We examined the expression of gE protein in DPV-infected cells using Western blotting, and analyzed the DPV gE gene transcription in DPV-infected cells using the real time PCR and RT-PCR.

## Results

### Cloning of DPV gE gene and the correct recombinant plasmid

**Figure 1:**
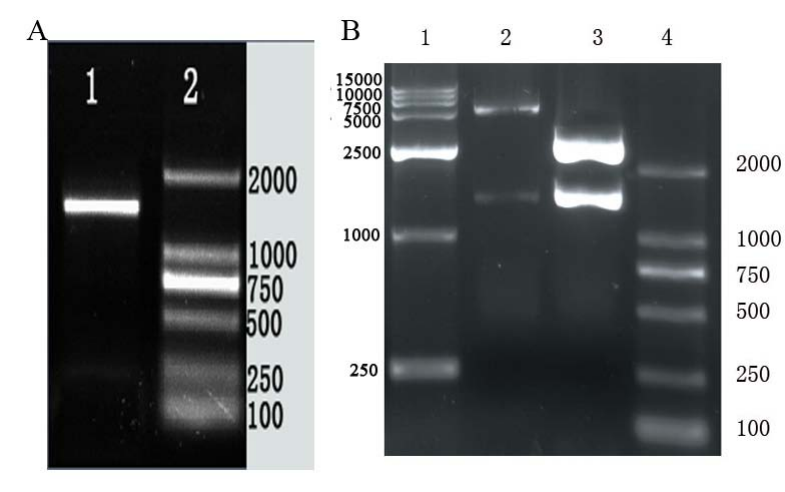
**PCR amplification of DPV gE gene and identification of the recombination vector**. A. Result of PCR amplification for DPV gE gene. Lane 1, the amplified product of DPV gE (about 1490bp); Lane 2, DNA marker 2000; B. Identification of the recombination vector pMD18/DPV-gE and pET32a/DPV-gE by restriction enzymes digestion. Lane 1, DNA marker 15000; Lane 2, the recombinant plasmid pET32a/DPV-gE was digested with EcoRI and XhoI (the PCR products with 1490 bp and the pET32a vector about 5,900bp) Lane 3, the recombinant plasmid pMD18/DPV-gE was digested with EcoRI and XhoI (the PCR products with 1490 bp and the pMD18-T vector about 2,700bp); Lane 4, DNA marker 2000.

Using the primers of DPV gE gene and Duck plague virus DNA as template, about 1490bp DNA product (restrictive site 12 bp, protective base 5 bp, and coding sequence of gE 1473 bp) was amplified by PCR. It was verified by 1% agarose gel electrophoresis (Fig 1A). The PCR product of approximate 1490bp was inserted into the pMDl8-T vector, thus the correct combinant plasmid was constructed, designated as pMD18/DPV-gE, and identified by restriction enzyme digestion analysis (Fig 1B). The constructed pMD18/DPV-gE was cut with EcoRI and XhoI, and the insert was ligated into pET32a(+) vector precut with the same enzymes. The recombinant vector was confirmed by restriction enzymes analysis, and it was verified by 1% agarose gel electrophoresis (Fig 1B). It showed that the expression plasmid pET32a/DPV-gE was successfully constructed.

### Expression and purification of the gE recombinant protein

**Figure 2:**
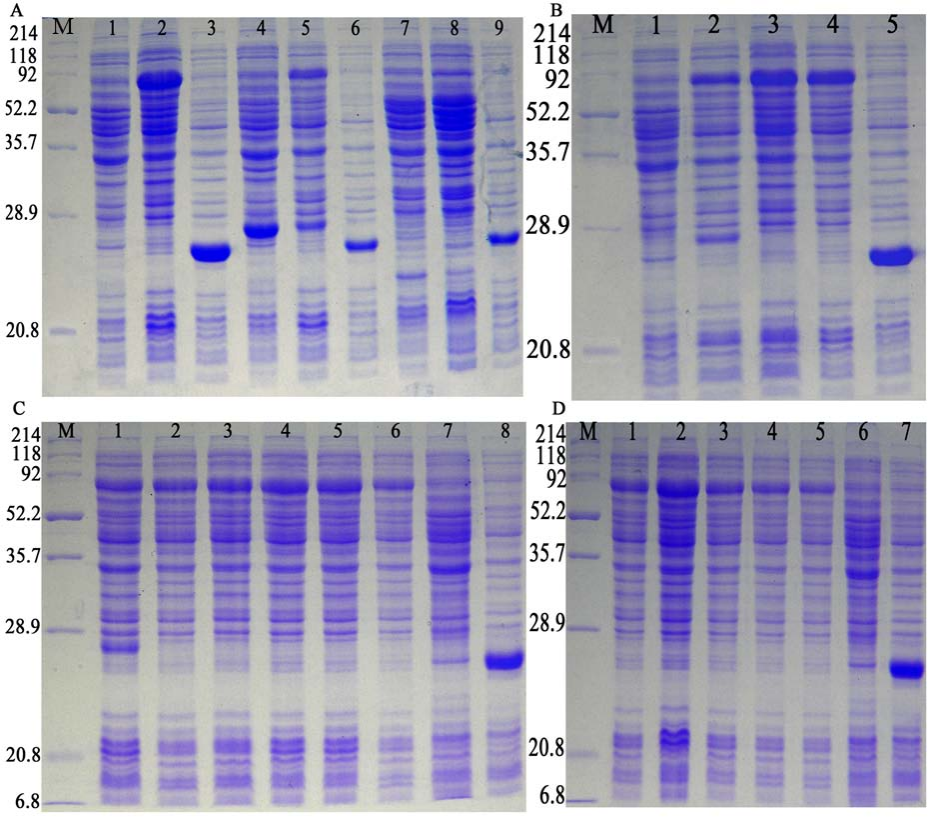
**The optimization analysis of the expression conditions of the pET32a/DPV-gE protein**. A. the pET32a/DPV-gE protein was expressed in E. coli Rosseta, BL21(DE3), BL21(pLysS) host strains. M: Protein molecular weight marker; Lane 1, the pET32a/DPV-gE before induction in E. coli Rosseta; Lane 2, the pET32a/DPV-gE after induction in E. coli Rosseta (about 74 kDa); Lane 3, the pET-32a(+) after induction in E. coli Rosseta (about 27 kDa); Lane 4, the pET32a/DPV-gE before induction in E. coli BL21(DE3); Lane 5, the pET32a/DPV-gE after induction; Lane 6, the pET-32a(+) after induction; Lane 7, the pET32a/DPV-gE before induction in E. coli BL21(pLysS); Lane 8, the pET32a/DPV-gE after induction; Lane 9, the pET-32a(+) after induction; B. Effect of different temperature of the pET32a/DPV-gE protein in Rosseta. M: Protein marker; Lane 1, the pET32a/DPV-gE before induction in E. coli Rosseta; Lane 2-4, the pET32a/DPV-gE after induction at 25, 30, and 37°C; Lane 5, the pET-32a(+) after induction; C. Effect of different time of the pET32a/DPV-gE protein in Rosseta. M: Protein marker; Lane 1-6, the pET32a/DPV-gE protein was expressed respectively for 2, 3, 4, 4.5, 5, and 6 h after induction with 0.2 mM IPTG; Lane 7, the pET32a/DPV-gE before induction; Lane 8, pET-32a(+) after induction; D. Production of recombinant plasmid pET32a/DPV-gE from Rosseta in different IPTG concentrations. M: Protein marker; Lane 1-5, the pET32a/DPV-gE protein was expressed respectively after induction with 0.1, 0.2, 0.4, 0.8, and 1.0 mM IPTG; Lane 6, the pET32a/DPV-gE before induction; Lane 7, the pET-32a(+) after induction.

To obtain a highly expressed level of pET32a/DPV-gE protein, the recombinant expression vectors pET32a/DPV-gE were transformed into the E.coli BL21(DE3), BL21(pLysS) and Rosseta expression host strains. And we tried optimizing expression conditions by using different temperatures (25, 30, 37°C), different IPTG concentrations (0.1, 0.2, 0.4, 0.8, 1.0 mM), and different incubation times (2, 3, 4, 4.5, 5, 6 h). We found that the expressed level of the pET32a/DPV-gE protein was better in Rosseta than in BL21(DE3) host strain, but the recombinant protein was not expressed in BL21(pLysS) (Fig 2A). And the expression level of the fusion pET32a/DPV-gE protein at 30°C was more than at 25°C and 37°C (Fig 2B). The different concentrations of IPTG showed apparent diversity in the expressed protein, and the expressed level of the protein was better after induction with 0.2 mM IPTG (Fig 2D). While the incubation time was increased, the expressed protein was increased too at first (Fig 2C), the highest level of expression was observed for 4.5 h after induction. Then the time was increased, the expressed protein was decreased. The results showed that the fusion pET32a/DPV-gE protein was highly expressed after induction at 30°C with 0.2 mM IPTG for 4.5 h in Rosseta. SDS-PAGE revealed a high level of expression of the approximately 74kDa recombinant protein was obtained.

**Figure 3:**
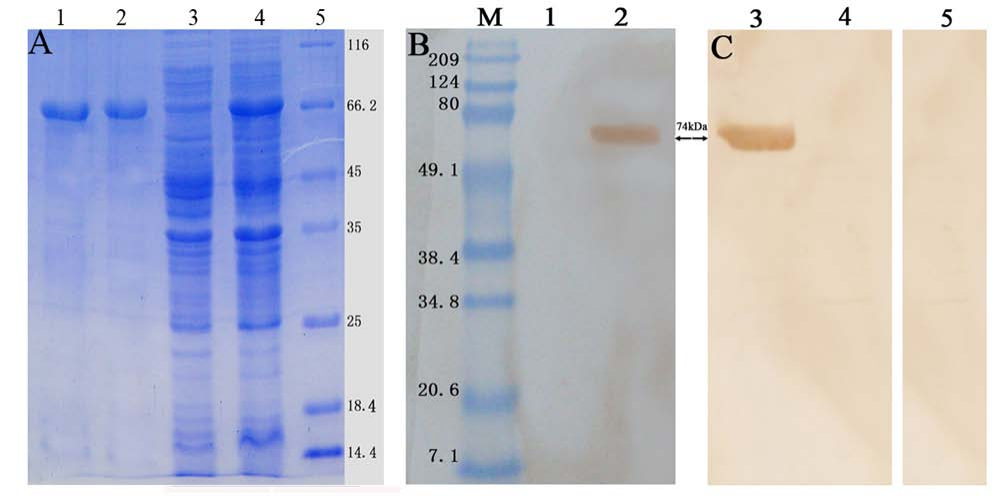
**SDS-PAGE of the purified pET32a/DPV-gE and Western blotting analysis**. A. The SDS-PAGE analysis of the purity of pET32a/DPV-gE. Lane 1-2, the recombinant protein was purified by the Ni^2+^-NTA agarose gel; Lane 3, the pET32a/DPV-gE before induction in E. coli Rosseta; Lane 4, the pET32a/DPV-gE after induction in E. coli Rosseta; 5: Protein marker. B. The immunogenicity of the recombinant gE protein was analyzed by western blotting with the rabbit anti-DPV IgG.. M: Prestained Protein Molecular Weight Marker; Lane 1, Expression of the recombinant plasmid pET32a/DPV-gE uninduced; Lane 2, Expression of the recombinant plasmid pET32a/DPV-gE induced by IPTG; C. The recombinant protein gE was recognized with the pET32a/DPV-gE antiserum. Lane 3, Expression of the recombinant plasmid pET32a/DPV-gE induced by IPTG; Lane 4, Expression of the recombinant plasmid pET32a/DPV-gE uninduced; Lane 5, the recombinant gE protein was analyzed with the pre-immune serum.

The fusion pET32a/DPV-gE protein was overexpressed with 0.2 mM IPTG in E. coli Rosseta and analyzed by SDS-PAGE. With purification using the Ni^2+^-NTA column by imidazole, the fusion pET32a/DPV-gE protein was separated from those of unwanted bacterial proteins. The protein yield (about 2.06 mg/ml) was measured by Bradford assay [24] and analyzed by SDS-PAGE (Fig 3A).

### Western Blotting

The immunogenicity of the recombinant protein gE was tested with the anti-DPV polyclonal IgG as the first antibody by western blotting analysis. The result indicated a single band at apparent molecular mass of 74 kDa region was obtained with the recombinant plasmid pET32a/DPV-gE in E. coli Rosseta, which was induced by IPTG (Fig 3B, Lane 2). However, the band was not detected without induction. (Fig 3B, Lane 1). And the recombinant protein gE was recognized with the pET32a/DPV-gE antiserum as the first antibody by western blotting analysis. The result showed a specific signal at about 74 kDa (Fig 3C, Lane 3), no positive signal was detected without induction (Fig 3C, Lane 4) and observed when using the pre-immune serum (Fig 3C, Lane 5).

### Dynamic proliferation of gE expression in DPV-infected cells

**Figure 4:**
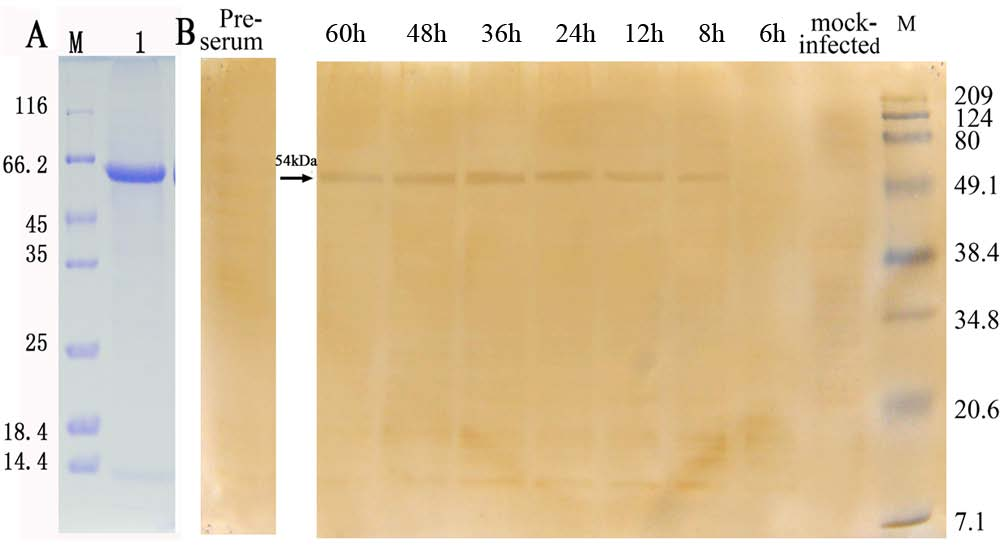
**Dynamic proliferation of gE protein in DPV-infected cells by western blotting**. A. The pET32a/DPV-gE antiserum was examined by SDS-PAGE; M: Protein Molecular Weight Marker; Lane 1: the pET32a/DPV-gE antiserum. B. The expression analysis of the gE gene product in DPV-infected DEFs with the pET32a/DPV-gE antiserum by Western blotting. DEFs were mock-infected or infected with DPV. The cells were harvested at 6, 8, 12, 24, 36, 48, and 60 h post-infection, and the lysates of DPV-infected cells at 36 h post-infection was analyzed by pre-serum. Molecular mass markers are shown on the right.

The dynamic proliferation of the gE protein expression in DPV-infected DEFs was analyzed at various times post-infection with the pET32a/DPV-gE antiserum by Western Blotting. The pET32a/DPV-gE antiserum was examined by SDS-PAGE (Fig 4A) and the reactivity and specificity of the pET32a/DPV-gE antiserum was performed. The results of Western Blotting showed that the gE protein was first detected at 8 h post-infection, the pET32a/DPV-gE antiserum was reacted with an approximate 54 kDa protein in lysates of DPV-infected cells (Fig 4B), and increased steadily, reaching a peak at 36 h post-infection, then the gE protein decreased gradually, the gE protein was least at 60 h post-infection. This band was not detected in mock-infected cells (Fig 4B), and the pre-immune serum did not recognize any proteins in lysates of DPV-infected cells at 36 h post-infection (Fig 4B). These results indicated that the pET32a/DPV-gE antiserum specifically detected the product of the gE gene.

### Intracellular localization of the gE product in DPV-infected cells

**Figure 5:**
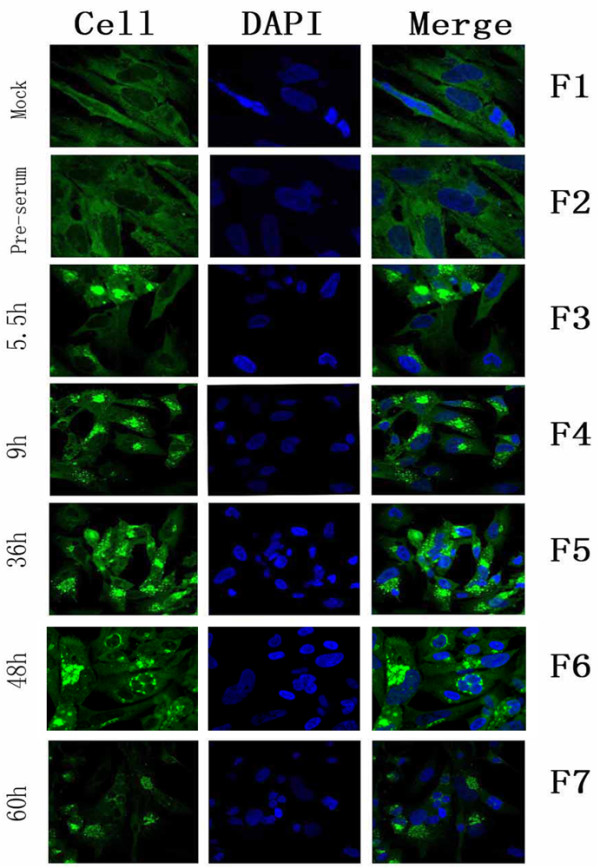
**Intracellular location and distribution of DPV gE analyzed by indirect immunofluorescence**. F1, Mock-infected cells were fixed; F2, DPV-infected were fixed at 5.5 h post-infection; F3-7, DPV-infected cells were fixed at 5.5 h, 9 h, 36 h, 48 h, and 60 h post-infection, the sample F2 was incubated with pre-immune serum, the other samples were incubated with the pET32a/DPV-gE antiserum. The merged fluorescence microscopy images of DEF are shown in panels F1 to F7 with high magnification (600×).

To confirm the intracellular localization of gE protein, indirect immunofluorescence studies were performed with the pET32a/DPV-gE antiserum. DEF cells were mock-infected or infected with DPV, and the infected samples were fixed in cold paraformaldehyde. The results showed the optimized conditions were as follows: the coverslips were fixed at 4°C overnight with 4% cold paraformaldehyde, and then treated with 3% BSA to block the nonspecific staining, the permeabilization time was with 0.2% (v/v) TrionX-100 in PBS for an additional 15 min at room temperature and the primary antibody was diluted 1:150 to incubate at 4°C overnight in the coverslips. As shown in Fig 5F3, the gE protein specific fluorescence was appeared in the cytoplasm region at 5.5 h post infection, and these fluorescence was clustered strongly and became stronger at 9 h post infection (Fig 5F4). At 36 h post infection (Fig 5F5), these fluorescence granules was detected widely distributed in the cytoplasm, and became more bigger and brighter. At 48 h post infection (Fig 5F6), the gE-specific fluorescence was detected especially in the juxtanuclear region of the cytoplasm, and gradually diminished. Then at 60 h post infection (Fig 5F7), the gE-specific fluorescence was more sparser and weaker following the cytoplasm disintegration in infected cells. No significant fluorescence was observed with pre-immune serum (Fig 5F2) or in mock-infected cells (Fig 5F1).

### Transcription analysis of the gE gene in DPV-infected cells

**Figure 6:**
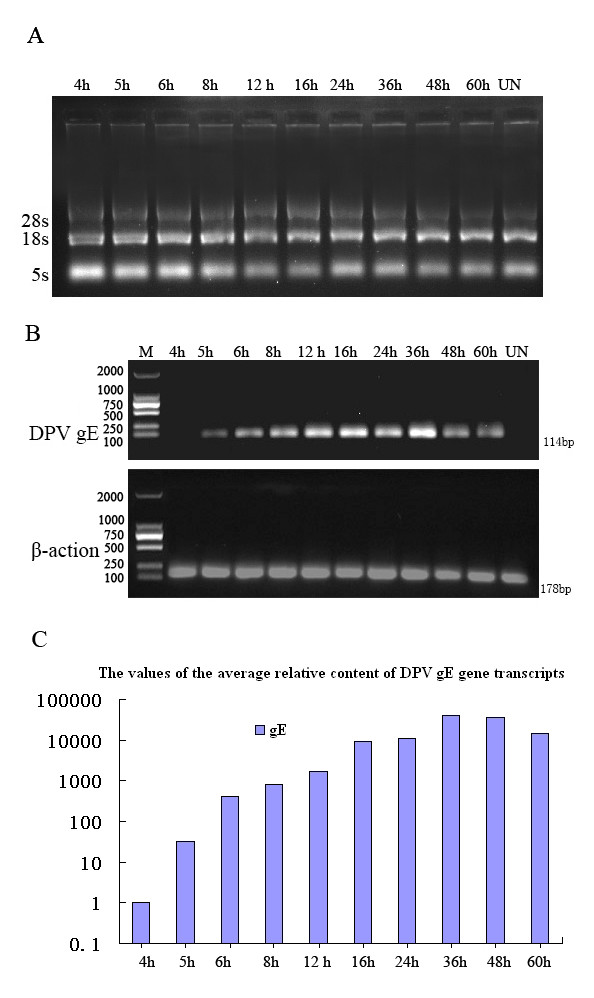
**The transcription analysis of DPV gE in mock-infected and DPV-infected DEFs**. A. Total RNA isolated from mock-infected and DPV-infected cells at various times post-infection was analysed using 1.0% agarose gel electrophoresis. B. The transcription analysis of DPV gE in infected DEFs with RT-PCR. The upper panel shows RNA from mock-infected DEFs (UN) and infected DEFs at different times (4, 5, 6, 8, 12, 16, 24, 36, 48, and 60 h) post-infection amplified by RT-PCR. M: molecular mass marker DL2000. The lower panel shows β-actin, which was run as an RNA-competence control. C. The transcription analysis of DPV gE in infected DEFs with. real time PCR. The DPV gE gene transcriptional expression was normalized to a reference gene (β-actin) and relative to the cells at 4 h post-infection. The average relative content of the DPV gE gene transcripts was calculated at 4, 5, 6, 8, 12, 16, 24, 36, 48, and 60 h post-infection using the 2^-ΔΔCt ^method.

The total RNA isolated from mock-infected and DPV-infected cells was verified by 1.0% agarose gel electrophoresis (Fig. 6A). The transcription of the DPV gE gene was analyzed by real-time quantitative PCR with SYBR Green I and reverse transcription-PCR (RT-PCR), the PCR samples amplified were detected by 1.0% agarose gel electrophoresis (Fig 6A). As shown in Fig 6B, the gE gene was detected at 5 h post-infection, and strongly increased at 36 h post-infection, then deceased at 48 h post-infection, and the DPV gE gene transcripts were not detected in mock-infected DEFs. The reference gene β-actin was no observable difference. The result of real-time quantitative PCR showed that the DPV gE gene transcripts were not detected in mock-infected control, and appeared as early as 4 h post-infection, then the content of transcripts increased steadily and reached a peak at 36 h post-infection, declining slowly thereafter. The average relative content of DPV gE gene transcripts were calculated using the 2^-ΔΔCt ^method. Fig 6C indicated the average relative content of DPV gE gene transcripts at 36 h post-infection was approximately 40,342 times that of the transcript at 4 h post-infection.

## Discussion

DPV gE is a typical membrane glycoprotein which spanned 490 amino acids. Computer analysis showed there were six putative N-glycosylation sites in DPV gE epitopes and there was an immunodominant region consisting of twenty-one distinct, conformation-dependent epitopes in DPV gE [10]. In this report, as the first step towards studying the properties and function of the gE protein. The PCR product of the gE was inserted into the vector pMD18-T, the recombinant plasmid pMD18/DPV-gE was confirmed by restriction digestion and DNA sequencing. The sequencing result showed that there were no nucleotide errors in the synthetic gE gene. This recombinant plasmid pMD18/DPV-gE could be used for further experiments to study the gE gene product.

We choosed the protocaryon expression vectors pET32a(+), which featured a high stringency T7 lac promoter, 6×His-tag, and thioredoxin, had been recognized as one of the most powerful tools for producing the recombinant proteins in E. coli [25]. The thioredoxin could not only reduce the digestion by bacterial proteases, but also promote the expression of the recombinant fusion protein [26]. The correct recombinant plasmid pMD18/DPV-gE was digested with EcoRI and XhoI, and the gE gene was directionally inserted in-frame downstream of the region encoding six histidine residues in the Escherichia coil expression vector pET32a(+). Expression of this fusion pET32a/DPV-gE protein is regulated by an IPTG-inducible lac operator and translation is expected to terminate at the stop codon of the gE gene. To obtain the highly expressed level of the fusion pET32a/DPV-gE protein as possible, the recombinant expression was transformed into E.coli BL21(pLysS), BL21(DE3) and Rosseta host cells, and optimized the condition for induction. Although there was 62 rare codons and 8 consecutive rare codons in gE ORF, which may influence the expression of the gE in vitro [27], the host bacteria Rosseta should impove the expression of the exogenous gene. The different temperatures, different IPTG concentrations, and different incubation times could effect the expressed level of the pET32a/DPV-gE protein. The result showed that the fusion pET32a/DPV-gE protein was highly expressed after induction at 30°C with 0.2 mM IPTG for 4.5 h in Rosseta.

We choosed the affinity purification using the immobilized metal affinity chromatography (IMAC) on nickel-nitrilotriacetic acid (Ni^2+^-NTA) affinity resin. The 6×His-Tag is very useful as a fusion partner for protein purification. 6×His-Tag fusion proteins can be affinity purified under denaturing conditions, which is particularly convenient for proteins expressed as inclusion bodies [11]. After elution with the equilibration buffer containing imidazole, a clear band corresponding to a molecular mass of about 74 kDa was seen on the SDS-PAGE gel following Coomassie blue staining. And Western blotting analysis showed that the fusion pET32a/DPV-gE protein was recognized by the rabbit anti-DPV IgG, it indicated that the protein had good immunogenicity, and the fusion pET32a/DPV-gE protein was used as antigen to produce the rabbit polyclonal antiserum specific for gE. And the fusion pET32a/DPV-gE protein was recognized with the pET32a/DPV-gE antiserum by Western blotting, these results indicated that the recombinant protein gE induced an immunological response and the pET32a/DPV-gE antiserum had a high level of specificity. In addition, the antiserum was examined to react specifically with apparent 54 kDa protein in DPV-infected cells in Western blotting experiments. These results indicated that the antiserum had a high level of reactivity and specificity, and the antiserum was used for further experiments to study the intracellular localization of the DPV gE.

The intracellular localization of DPV gE was examined by indirect immunofluorescence assay and confocal microscopy on DPV-infected DEFs. The data indicated that the protein was detected in the cytoplasm at 5.5 hours post-infection. During the IFA process, the permeabilization time, and the dilution concentration of the primary antibody were two significant factors, the permeabilization time influenced the pET32a/DPV-gE antiserum to penetrate into the cell sufficiently, and the dilution concentration of the primary antibody effected the dense of the gE-specific fluorescence. So we obtained the optimized conditions was with 0.2% (v/v) TrionX-100 in PBS for an additional 15 min at room temperature, and the primary antibody was diluted 1:150 to incubate with the cells at 4°C overnight.

DPV belonged to the Alphaherpesvirinae subfamily of herpesviruses, and possesed a lipidic envelope in which different glycoproteins of viral origin are embedded [28,29]. About the pathways of Alphaherpesviruses during their intracellular maturation, some reports supported that the nucleocapsids got transient envelops from the inner lamella of nuclear membrane, which would fuse with the membrane of the endoplasmic reticulum. The naked nucleocapsids were released into the cytosol, and they became enveloped during budding into cytosolic membraneous compartments, most probably trans-Golgi network [30,31]. Some studies had reported that the gE glycoprotein had also been detected in the cytoplasm of the HSV-1-infected cells, VZV-infected cells, and PRV-infected cells. In this report, the result revealed that the DPV gE was targeted to the cytoplasm of DPV-infected cells, similar to the gE homologous protein of HSV-1, VZV-1, and PRV [32-34], and suggested that DPV gE protein might serve similar functions with the gE homologous protein. And some reports had illustrated the role of Tyrosine-containing sorting motifs in regulating the intracellular traffic of membrane proteins [33,35]. The Tyrosine-containing sorting motifs usually consist of a tetrapeptide bearing the sequence YXXØ (Y is tyrosine, X is any amino acid, and Ø represents any hydrophobic residue) [36,37]. The DPV gE protein contained YGSY and YNSL in the cytoplasmic domain [10], we inferred that 2 motifs could mediate the intracellular traffic of DPV gE protein. The research will provide useful clues for further understanding the localization properties of the alphaherpesvirus gE homologs.

Currently, there is little information on the transcription and translation of DPV gE. We studied the transcription of the DPV gE gene using RT-PCR and real-time quantitative PCR. DPV gE earliest transcripts were detected at 5 h post-infection by RT-PCR, and markedly increased at 36 h post-infection. The analysis of real-time quantitative PCR showed that DPV gE earliest transcripts can be detected at 4 h post-infection, and the average relative content of DPV gE transcripts at 36 h post-infection was approximately 40,342 times that of the transcript at 4 h post-infection. It indicated that real-time quantitative PCR was more sensitive than the conventional RT-PCR. We studied the dynamic proliferation of the gE protein expression in DPV-infected DEFs using Western blotting and indirect immunofluorescence assay. The DPV gE protein was first observed at 8 h post-infection, with maximal amounts at 36 h post-infection, and then declining gradually. However, the indirect immunofluorescence assay was highly sensitive. The gE protein specific fluorescence was observed firstly in the cytoplasm region at 5.5 h post infection and increased gradually. These results demonstrated that the accumulation of the gE protein occurred at the late stage of infection. Kocan R M [38] reported that DPV had a latent period of 6 hours and a maximum virus titer reached at 36 hours in DPV-infected cells at a multiplicity of 2 PFU/cell. However, the number of DPV-infected effected the latent period in virus replication. In this report, the cells were infected with DPV at a multiplicity of 5 PFU/cell, it inferred that the latent period of DPV would be less than 6 h, and the result showed that the gE was detected at 4 h post-infection by real-time quantitative RT-PCR, Guo [39] had reported that real-time PCR assay for the detection of DPV could detected the 1.0 × 10^1 ^copy, so it indicated that gE begun to transcribe at 4 h post infection and would take part in assembling with the envelope to form mature DPV virions.

## Conclusions

In conclusion, the DPV gE gene has been successfully expressed in a prokaryotic expression system, and we present the basic characteristics of DPV gE product. The immunofluorescence studies showed that gE mainly localized in the cytoplasm, and DPV gE might share similar functions with its HSV-1, VZV-1, and PRV homolog gE. The real time PCR, RT-PCR, and Western blotting analysis indicated that the accumulation of DPV gE protein was observed at the late stage of infection. These results were especially helpful for the functional analysis of the DPV gE protein.

## Materials and methods

### Materials

DPV CHv strains and the rabbit anti-DPV were provided by Key Laboratory of Animal Disease and Human Health of Sichuan Province. The expression vector pET32a(+) and the host strain Escherichia coli BL21(DE3), BL21(pLysS) and Rosseta were purchased from Novagen. Primers were synthesized at TaKaRa (Dalian, China). Restriction enzymes, EcoRI and XhoI, pMD18-T vector, the Total RNA Isolation System and RNase-free DNase I were purchased from TaKaRa Biotechnology Co. Ltd. The Gel extraction kit purification, and the real-time PCR Master Mix SYBR Green I were purchased from Tiangen Biotechnology Co. Ltd. Horseradish peroxidase (HRP)-conjugated goat anti-rabbit IgG, the fluorescein isothiocyanate-conjugated secondary antibody (goat anti-rabbit) and DAB (3'3'-Diaminobenzidine tetrahydrochloride peroxidase) were from Beijing Zhongshan Co. Ltd.

Duck embryo fibroblasts (DEF) were cultured in MEM medium (Gibco-BRL) supplemented with 10% fetal bovine serum (FBS) (Gibco-BRL) at 37°C. For virus infection, MEM medium supplemented with 2-3% FBS was used [40].

### Primer Design and PCR Amplification of the gE Gene

The coding regions of gE gene was amplified by PCR using the primers. The forward primer (P1) is 5'-**CGGAATTC**ATGATGGTTACTTTTATATCTACAG-3', containing a EcoRI site, protective base (underlined) and the first 25nt of the gE ORF, and the reverse primer (P2) 5'-**CCGCTCGAG**TCAGATGCGGAAACTAGA-3', with a XhoI site, protective base (underlined) and the last 18nt of the gE.

The PCR reagent was composed of 2.5 μl of 10 × reaction buffer, 2.0 μ1 dNTPs (2.5 mM for each of the four dNTPs), 1.0 μl of each primer (20 pM each), 2.0 μl DNA template, 2.0 μl MgCl_2_, 0.25 μl Taq DNA polymerase (5U/μl), Sterile water was added into the mixture to 25 μl. Reactions were performed at 95°C for 5 min, followed by 30 cycles of 94°C for 45 s, 58°C for 45 s and 72°C for 1.5 min, followed by 72°C for 10 min. The amplified product was verified by 1% agarose gel electrophoresis and analyzed using gel imaging system (Bio-Rad, USA).

### Cloning of the gE Gene and Construction of recombinant expression vector

The PCR amplified product of the gE gene was purified by the Gel Extraction kit according to the manufacturer's instructions. The purified product was ligated into pMD18-T vector which was an AT cloning vector at 16°C overnight using T4 DNA ligase. Competent E. coli DH_5α_cells were transformed with the ligation mixture by the heat shock method. The cells were cultured at 37°C on Luria-Bertani broth plates containing 100 mg/ml ampicillin for 16 h. Then the recombinant plasmid was confirmed by restriction enzyme digestion (EcoRI and XhoI). The correct recombinant plasmid was sent to Dalian TAKARA Biotechnology Co. (China) for sequencing.

The correct recombinant vector was named as pMD18/DPV-gE. Subsequently, the constructed pMD18/DPV-gE was cut with EcoRI and XhoI, and the insert was subcloned into the pET32a(+) expression vector [41] precut with the same enzymes. Competent E. coli DH_5α_cells were transformed with the ligation product. Cells were cultured overnight at 37°C on Luria-Bertani broth plates containing 100 mg/ml ampicillin. The subclones were verified by restriction analysis (EcoRI and XhoI). Escherichia coli BL21(pLysS), BL21(DE3) and Rosseta cells were individually transformed with the positive recombinant plasmid and used for protein expression.

### Expression and Purification of the recombinant protein

Expression of this fusion protein was regulated by an IPTG-inducible lac operator sequence and a phage T7 promoter. To obtain as much fusion protein as possible, we transformed the recombinant expression into E.coli BL21(DE3), BL21(pLysS) and Rosseta host cells, and optimized the condition for induction. Once an optical density at 600 nm (OD 600nm) of the cultures reached about 0.5, the bacterial culture was induced with different concentrations of IPTG (0.1-1.0 mM) or allowed to grow for 2-6 h at 25, 30, 37°C. The cells were harvested by centrifugation at 10,000 rpm/min for 5 min, and the cell lysate was lysed in SDS sample buffer (0.5 M Tris-HCl, pH 6.8, 50% glycerol, 10% SDS, and 0.05% bromophenol blue, with 100 mM DTT). The pellet was heated at 95°C for 10 min, and analyzed by SDS-PAGE using 12% polyacrylamide gel. The uninduced control culture and the vector control culture were analyzed in parallel.

Recombinant pET32a/DPV-gE protein was purified under denaturing condition using the immobilized metal affinity chromatography (IMAC) on nickel-nitrilotriacetic acid (Ni^2+^-NTA) affinity resin (Bio-Rad). The induced cells were centrifuged at 10,000 rpm/min for 10 min, and lysed in 20 ml 20 mM Tris-HCl pH 8.0 containing 1.0 mg/ml lysozyme at -20°C overnight. The cell lysate was clarified by centrifugation at 10,000 rpm/min for 20 min at 4°C and the supernatant was discarded, after it was disrupted by an ultrasonic cell disrupter with pulses of 200 W for 30 s intermittence 10 times. The pellet of the inclusion bodies was resuspended in 20 ml cold washing buffer (20 mM Tris-HCl, 2% Triton X-100 (v/v), pH 8.0) under constant stirring for 10 min, then followed by centrifugation at 10,000 rpm/min for 10 min at 4°C, and the above steps were repeated once. Finally, the pellet was solubilized in denaturing buffer (8 M urea, 100 m M NaH_2_PO4, 10 mM Tris-HCl, pH 8.0). Denatured soluble protein was loaded on the column, and the 6×His-Tag recombinant protein was eluted from the column by 100 ml linear gradient equilibration buffer containing 20-250 mM imidazole, with protein purification system (Bio-Rad, USA). Bound protein fractions were pooled, dialyzed, and concentrated, and the expression yield was analyzed by Bradford assay [24].

### Western Blot Analysis

The pET32a/DPV-gE protein separated on 12% SDS-PAGE gel was transferred to the polyvinylidene difluoride (PVDF) membrane. The membrane was incubated with blocking buffer containing 3% bovine serum albumin (BSA) in TBS (50 mM Tris-HCl, 150 mM NaCl, pH 7.5) for 1 h at 37°C. Subsequently, the membrane was incubated with the serum of the rabbit anti-DPV (diluted 1:200) for 1 h at 4°C overnight, and washed 3 times for 5 min each with TBS containing 0.05% Tween-20 (TBST), and incubated for 2 h with HRP-conjugated goat anti-rabbit IgG (diluted 1:10000). The membrane was again washed with TBST, and developed with substrate solution (DAB 3'3'-Diaminobenzidine tetrahydrochloride peroxidase) for 3 min at 37°C. Finally, the reaction was stopped by the addition of distilled water to strips.

### Generation of polyclonal antisera in the rabbits

The purified denatured protein was done by gradient dialysis in 0.85% NaCl solution containing 6, 5, 4, 3, 2 M urea, and changed 3 times over 1 day at 4°C in each solution. Also, aggregation was removed by centrifugation and the supernatant was collected as soluble refolded protein. For the preparation of polyclonal antibodies, the rabbits were immunized intradermally with purified recombinant protein (0.75 mg per rabbit with the addition of Freud's complete adjuvant), after a week, the rabbits were immunized intradermally with purified recombinant protein (1.00 mg per rabbit with the addition of Freud's incomplete adjuvant), the rabbits were immunized subcutaneously with purified recombinant protein (1.00 mg per rabbit with the addition of Freud's incomplete adjuvant) 7 days after the second injection, after 7 days, the rabbits were injected by the vein of the edge of the ears with 0.5 mg per rabbit. Sera were collected 17 days after fourth injections, and stored at -80°C until further use. Control pre-immune serum was obtained before the first injection. The purified pET32a/DPV-gE antiserum was obtained by purification using ammonium sulfate precipitation and High-Q anion-exchange chromatography [42]. Western blottiong analysis was conducted to examine the reactivity and specificity of the pET32a/DPV-gE antiserum.

### The expression of gE protein in DPV-infected cells

DEFs were either mock-infected or infected with DPV at a multiplicity of 5 PFU per cell, and harvested at 6, 8, 12, 24, 36, 48 and 60 h post-infection. Cells were lysed in SDS sample buffer, the pellet was heated at 95°Cfor 10 min and size-separated by electrophoresis on 12% SDS-containing polyacrylamide gels followed by transfer of protein onto PVDF membrane. After transferring, the membrane was incubated at 37°C for 60 min with blocking buffer (3% bovine serum albumin in PBST) at 37°C, and subsequently incubated with the purified pET32a/DPV-gE antiserum (diluted 1:150) for 1 h at 37°C. The membrane was washed three times with PBST (PBS plus 0.1% Tween-20), 10 min each and then incubated with horseradish peroxidase-link sheep anti-rabbit IgG (1:10000) for 1 h at 37°C. Following three 10 min washes with PBST, DAB (3'3'-Diaminobenzidine tetrahydrochloride peroxidase) substrate was used as a substrate to visualize the reaction result according to manufacturer's instructions (Beijing Zhong Shan Co. Ltd., China).

### Intracellular localization of the gE protein in DPV-infected cells

To characterize the intracellular localization of gE protein, immunofluorescent microscopy analysis was employed with the anti-pET32a/DPV-gE polyclonal antibody as described previously [43]. DEFs grown on glass coverslips were infected with DPV at a multiplicity of 5 PFU/cell. At different times (5.5, 9, 36, 48, and 60 h) post-infection, the cells were collected, and the mock-infected cells were collected. After washing (PBS), the coverslips were fixed immediately for 4% paraformaldehyde for 3 h at 4°C. After permeabilization (PBS, 0.1% triton X-100 for 15 min) and blocking (3% bovine serum albumin in PBS-T for at 4°C overnight), the coverslips were incubated with the pET32a/DPV-gE antiserum for 2 h at 37°C. Following incubation with the primary antibody, the coverslips were washed 3 times in PBS containing 0.2% Tween-20 and stained with fluorescein isothiocyanate-conjugated secondary antibody (goat anti-rabbit, Beijing Zhongshan Co. Ltd) for 30 min. The coverslips were again washed 3 times and stained with 4'6'-diamidino-2-phenylindole (DAPI) for 10 min. To obtain the optimized conditions, the fixed temperature (4°C, 37°C) and time (30 min, 1 h, 4 h and overnight), permeabilization time (5 min, 15 min, 30 min), the blocking buffer (3% bovine serum albumin, 5% bovine serum albumin), the dilution concentration of the primary antibody (1:50, 1:100, 1:150) and incubation time (37°C 30 min, 60 min, 90 min and 4°C overnight) were performed. Finally, the coverslips were mounted onto glass slides with a drop of mounting medium (PBS containing 90% glycerol), and analyzed with Confocal laser scanning microscopy (CLSM, Nikon, Japan).

### RNA expression of DPV gE in DPV-infected cells

DEFs were infected with DPV at a multiplicity of 5 PFU per cell. To examine the gE transcription in infected cells in vitro, the total RNA was isolated from mock-infected or DPV-infected cells at different times (4, 5, 6, 8, 12, 16, 24, 36, 48 and 60 h post-infection) by using the Total RNA Isolation System (Takara), and detected by 1.0% agarose gel electrophoresis. The cell volume equivalent amount of total RNA (15 μl) was digested by the RNase-free DNase I (Takara) to eliminate contamination of chromosomal DNA. The concentration of RNA was determined by measuring A260, and the purity was checked by the A260/A280 ratio (greater than 1.8), 100 ng RNA was used as template for RT-PCR. According to the manufacturer's instructions (TaKaRa), the RT reaction was performed in a 10 μl reaction volume.

Based on the nucleotide sequence of the DPV gE gene, the forward primer is 5'-AAAATAACATCGTGGGC-3', and the reverse primer is 5'-TTCGGTAGACTTTAG CATC-3'. Using duck β-actin as the reference gene, the forward primer is 5'-CCGGGCATCGCTGACA-3', and the reverse primer is 5'-GGATTCATCATACTC CTGCTTGCT-3'. RT-PCR was performed in a volume of 25 μl containing 1.0 μl of the forward primer (10 pmol/L), 1.0 of the reverse primer (10 pmol/L), 1.0 μl cDNA template, 12.5 μl PCR Master Mix, and 9.5 μl water. β-actin mRNA expression was determined using the same amount of cDNA as an RNA-competence control. Real time PCR was performed in a volume of 25 μl containing 1.0 μl of the forward primer (10 pmol/L), 1.0 of the reverse primer (10 pmol/L), 1.0 μl cDNA template, 12.5 μl real-time PCR Master Mix SYBR Green I, and 9.5 μl water (all reagents were purchased from TaKaRa). All reactions were performed in triplicate and in at least two independent reactions, and the average relative content of DPV gE gene transcripts was calculated using the 2^-ΔΔC t ^method [44].

## Competing interests

The authors declare that they have no competing interests.

## Authors' contributions

HC carried out most of the experiments and wrote the manuscript. ACC and MSW critically revised the manuscript and the experiment design. DKZ, RYJ, FL, ZLC, QHL, XYC, YZ helped with the experiment. All of the authors read and approved the final version of the manuscript.
